# The Relationship between Depressive Symptoms, Loneliness, Self-Control, and Gaming Disorder among Polish Male and Female Gamers: The Indirect Effects of Gaming Motives

**DOI:** 10.3390/ijerph191610438

**Published:** 2022-08-22

**Authors:** Andrzej Cudo, Marcin Wojtasiński, Przemysław Tużnik, Agnieszka Fudali-Czyż, Mark D. Griffiths

**Affiliations:** 1 Institute of Psychology, The John Paul II Catholic University of Lublin, al. Racławickie 14, 20950 Lublin, Poland; 2 Institute of Psychology, The Pedagogical University of Krakow, ul. Podchorążych 2, 30084 Krakow, Poland; 3 International Gaming Research Unit, Psychology Department, Nottingham Trent University, Nottingham NG1 4FQ, UK

**Keywords:** gaming, internet gaming disorder, gaming motives, depression, loneliness, self-control, gender differences

## Abstract

The present study analyzed the relationship between gaming disorder (GD), motives for gaming, and psychological problems in daily life (i.e., depression, loneliness, and self-control deficits) among Polish gamers. More specifically, the purpose of the present study was to analyze the indirect effects between GD and psychological problems in daily life via motives for gaming among male and female gamers. Additionally, the present study examined gender differences in motives for gaming and the relationship between the analyzed variables. The sample comprised 652 gamers (233 females; M = 28.77 years; SD = 7.18; age range: 18–48 years). The nine-item Internet Gaming Disorder Scale-Short Version (IGDS9-SF) was used to assess GD. The motives for gaming were assessed using the Motives for Online Gaming Questionnaire (MOGQ). The nine-item Patient Health Questionnaire (PHQ-9) was used to assess depressive symptoms, and the Brief Self-Control Scale (BSCS) was used to assess self-control. Loneliness was assessed using the De Jong Gierveld Loneliness Scale. In order to examine the relationship between the study variables, path analysis and indirect effects analysis were performed among both male and female gamers. The present study showed that depressive symptoms and self-control exerted a significant indirect effect on GD via escape and fantasy motives for gaming. Additionally, there was an indirect effect between depressive symptoms and GD via social motives for gaming. However, these indirect effects were found among male gamers only. The present study also showed gender differences in all analyzed gaming motives except escape (males scoring higher on all of them) and in the relationship between depressive symptoms and escape. These findings contribute to a better understanding of GD development mechanisms, which are associated with psychological problems in daily life.

## 1. Introduction

Technology has undoubtedly dominated everyday life and has developed markedly in recent years. It is important that despite the unquestionable advantages of this development, attention to the potential risks should always be kept in mind. Computers, smartphones, and the internet make it possible for individuals to experience enjoyment and entertainment, in addition to facilitating work and providing a wide variety of real-time information [1]. This interaction is particularly visible in a specific type of entertainment—videogames. Videogames have been defined as “a mode of interaction between a player, a machine with an electronic visual display, and possibly other players, that is mediated by a meaningful fictional context, and sustained by an emotional attachment between the player and the outcomes of his/her actions within this fictional context” [2] (p. 253). Currently, it is estimated that the number of online gamers worldwide exceeds one billion individuals and could reach 1.3 billion by 2025 [3].

Poland (where the present study was carried out) has witnessed a strong growth in gamers, with 17.6 million individuals being gamers and females accounting for 35% of all gamers [4]. For years, the question ‘why do individuals engage in video gaming?’ has interested researchers. The frequently explored study areas include the advantages and disadvantages that gaming can bring [5,6,7]. Another area of study is the reasons as to why some gamers can play videogames adaptively while others develop gaming disorder (GD). Consequently, the present study examined the relationship between GD, motives for gaming, and psychological problems in daily life including depressive symptoms, loneliness, and self-control deficits (see [8,9]). More specifically, the purpose of the present study was to analyze the indirect effects between GD and psychological problems in daily life via motives for gaming. Additionally, considering the small number of studies on gaming among female gamers see [10,11], the present study’s purpose was also to examine female motives for gaming and compare these motives to those of male gamers.

Considering the increasing number of empirical studies regarding the problematic and potentially addictive effects of videogames on their users [12], GD is an issue that has been particularly explored in the past two decades. Additionally, GD criteria are included in two of the most important manuals for the classification of the disorder: (i) the fifth edition of the *Diagnostic and Statistical Manual of Mental Disorders* (DSM-5) [13] and (ii) the eleventh revision of the *International Classification of* Diseases (ICD-11) [14]. The APA introduced ‘Internet Gaming Disorder’ (IGD) in Section III of the DSM-5 (Condition for Further Study) because the disorder is tentative and requires further research [13]. IGD is said to be present when at least five of the nine following diagnostic criteria occur within the past 12 months: (i) preoccupation with gaming; (ii) withdrawal symptoms when gaming is taken away; (iii) increase in gaming over time (i.e., tolerance); (iv) unsuccessful attempts to control gaming; (v) loss of interests in previous hobbies and entertainment as a result of (with the exception of) gaming; (vi) continued excessive gaming despite the existence of psychosocial problems; (vii) deceiving family members, therapists, or others regarding the amount of gaming; (viii) gaming to escape or relieve a negative mood (e.g., feelings of helplessness, guilt, anxiety); and (ix) jeopardizing or losing a significant relationship, job, or educational or career opportunity because of gaming [13]. The ICD-11 proposed that GD is “characterised by a pattern of persistent or recurrent gaming behavior (…) manifested by: (1) impaired control over gaming (…); (2) increasing priority given to gaming to the extent that gaming takes precedence over other life interests and daily activities; and (3) continuation or escalation of gaming despite the occurrence of negative consequences” [14]. 

Based on the Interaction of Person–Affect–Cognition–Execution (I-PACE) model [8,9], GD may be viewed as a subtype of addictive behavior [9]. According to the I-PACE model, the development of behavioral addiction is associated with the interaction between individuals’ predisposing variables (e.g., genetics, early childhood experiences, psychopathology, temperamental features, general coping style, specific needs, specific motives, specific values) and situations that an individual encounters in their life. This interaction may lead to gratification and/or compensation experiences associated with the specific behavior [9]. As a result, an individual may maintain behaviors (e.g., gaming) where overuse may have negative consequences for them (e.g., decreased academic performance, dismissal from a job). Additionally, Brand et al. [9] highlight the relevance of control mechanism deficits in behavioral addiction development. More specifically, individuals who have difficulty controlling their responses to triggers (e.g., game-related stimuli) can more easily become addicted to the behavior (e.g., gaming). Consequently, in terms of factors that contribute to the development of behavioral addictions, including GD, Brand et al. [8,9] emphasize the importance of depression, self-control deficits, and loneliness as predictors of this type of addiction.

### 1.1. Depressive Symptoms, Loneliness, Self-Control, and Gaming Disorder

Previous research [15,16,17,18] has shown an association between depressive symptoms and GD. More specifically, higher depressive symptoms have been positively associated with higher GD symptoms. It should be noted that, according to the socio-cognitive model of unregulated use of media [19], GD may result from a lack of self-regulation. In this context, the ability to self-regulate may be reduced by depression. Considering the socio-cognitive model of unregulated media use, it can be assumed that greater knowledge of videogames and higher expectations of gaming activity effects may be associated with stronger gaming compulsive habits [19]. According to the I-PACE model [8,9], depressive symptoms can be important factors associated with the likelihood of developing addictive gaming. However, longitudinal studies have indicated that depression may be an antecedent of GD [20,21] and that depression and GD have a reciprocal relationship [22,23].

Previous studies [24,25] have also shown the negative relationship between self-control and GD. In this context, Cudo et al. [24] reported the negative relationship between GD and self-control associated with the ability of efficient motivation induction and persistence in achieving a higher-order goal. Additionally, they reported differences between male and female gamers in the case of the relationship between GD and self-control associated with inhibition and adjournment (i.e., suppression or delay of unwanted or improper actions which may contribute to the failure of goal-directed behavior). More specifically, the negative relationship between these variables only occurred among male gamers. 

Moreover, Mills and Allen [25] reported a relationship between low self-control and GD, weekly gaming time, and less adaptive videogame playing motivations. Additionally, they showed an indirect effect between self-control and GD via amotivation and introjected regulation. In this context, introjected regulation is related to internal pressures to engage in videogame playing. In contrast, amotivation is related to engaging in videogames despite not wanting to play them. In this context, according to the I-PACE model [8,9], self-control deficits can be important factors in increasing the likelihood of developing addictive gaming. In particular, these deficits can increase the difficulty of inhibiting triggers.

Pontes et al. [18] reported a positive relationship between loneliness and GD. Additionally, in longitudinal studies, Dutch [26] and Norwegian [23] studies found that loneliness was both an antecedent and a consequence of GD. Consequently, loneliness may be an important factor contributing to the development and maintenance of GD. However, Cudo, Kopiś, and Zabielska-Mendyk [27] reported that the relationship between loneliness and GD was fully mediated by personal distress, defined as “the self-oriented feelings of personal anxiety and unease in tense interpersonal settings” [28] (p. 114). In this context, according to the compensatory internet use model [29], it is postulated that gamers who cannot manage real-life situations or have difficulties with meeting their needs (e.g., need for social contact) can use the videogame in order to moderate negative emotions and compensate their needs in virtual reality.

Taken together, depressive symptoms, loneliness, and self-control deficits are important factors associated with GD development (see [8,9,19]). However, the relationship between these factors and GD is not fully understood. More specifically, it seems important to answer the question of what motives for gaming may lead individuals with depressive symptoms, loneliness, and/or low self-control to addictive videogame use. A more detailed understanding of these relationships may contribute to developing more accurate methods for GD prevention and treatment.

### 1.2. Gaming Motives and Gaming Disorder

Individuals use videogames for a variety of motives. However, it is possible to classify these motives into seven dimensions: (i) social-gaming as a source of pleasure from contact with other gamers and from being with others while gaming; (ii) escape-gaming as an escape from reality, especially from problems in the real world; (iii) competition-gaming as an opportunity to compete with others gamers and beat them in order to have a sense of achievement; (iv) coping-gaming as a way to cope with stress and aggression, and to improve mood; (v) skill development-gaming as an opportunity to improve coordination, concentration, and other cognitive skills; (vi) fantasy-gaming as an opportunity to break away from one’s usual identity, try new identities in another world, and try things that cannot be done in real life; and (vii) recreation-gaming as a source of fun and relaxation [30]. Previous research has shown that GD was especially positively associated with escape, competition, and coping motives [31,32,33,34] and negatively associated with skill development and recreation [31,34]. Additionally, Kircaburun et al. [32] reported that the escape motive for gaming was a mediator in the relationship between emotional intelligence and GD. The escape and fantasy motives for gaming mediated the relationship between GD and the dark tetrad personality traits such as Machiavellianism, narcissism, and sadism [35]. 

Maroney et al. [36] also reported that the escape motive for gaming was a mediator in the relationship between depression and GD and between loneliness and GD. Moreover, Király et al. [31] reported that escape and competition motives for gaming were a mediator in the relationship between general distress and GD among male and female gamers. Montag et al. [16] showed that escape, competition, skill development, and recreation motives for gaming mediated the relationship between depression and GD. Additionally, they reported that escape, competition, coping, skill development, and recreation motives for gaming mediated the relationship between loneliness and GD and between attention problems and GD. Therefore, it appears that escape, competition, skill development, and recreation are important mediators of the relationship between GD and the psychological problems in daily life such as depressive symptoms, loneliness, and self-control deficits. However, considering the findings of previous studies showing gender differences in GD prevalence [12], GD risk factors [10,24,37,38], and gaming motives [31,34], gender is an important variable that cannot be ignored.

### 1.3. Gender Perspectives

The results of a meta-analysis conducted by Stevens et al. [12] found that GD occurs more often in males than in females. However, it should be noted that female gamers have different gaming characteristics compared to male gamers. More specifically, McLean and Griffiths [39] reported that female gamers can experience anxiety and loneliness due to a lack of social support during gaming. Additionally, female gamers reported the experience of using different strategies to cope with harassment from males while gaming. Consequently, female gamers may have different motivations for gaming than male gamers. In this context, Király et al. [31] showed that female gamers had higher levels of escape, fantasy, recreation, and social motives for gaming than male gamers. In contrast, they reported that male gamers had higher competition motives for gaming than female gamers. Additionally, there was a gender difference in the relationship between escape motives and GD. More specifically, female gamers presented a stronger relationship between these variables than male gamers [31].

However, Laconi, Pirès, and Chabrol [34] showed that male gamers had a higher level of motives for gaming, such as social, competition, skill development, fantasy, and recreation, than female gamers. Additionally, they [34] reported that GD was positively associated with escape and coping motives for gaming among male gamers. For female gamers, GD was positively related to escape and competition motives for gaming and negatively related to skill development. Consequently, female gamers may show a different relationship pattern between depressive symptoms, loneliness, self-control, and GD via motives for gaming than male gamers. However, the exact pattern of these relationships has not been entirely clarified (cf. [31,34]).

### 1.4. The Present Study

Based on the literature reviewed, the present study analyzed the relationship between GD and psychological problems in daily life such as depressive symptoms, loneliness, and self-control deficits (see [8,9]) via motives for gaming. In this context, taking into account the results of previous studies, e.g., [16,31,32], it is hypothesized that there will be an indirect effect of the escape (H_1_), competition (H_2_), skill development (H_3_), and recreation (H_4_) motives for gaming in the relationship between GD and the psychological problems such as depressive symptoms, loneliness, and self-control deficits. Additionally, the present study analyzed gender differences in motives for gaming and the relationship between these motives and GD. Consequently, considering the differences between female and male gamers, e.g., [31,34,39], it is hypothesized that there will be gender differences in gaming motives (H_5_). Additionally, considering gender differences in the relationship between GD and motives for gaming [34], it is hypothesized that there will be a gender difference in the relationship between psychological problems in daily life and GD via motives for gaming (H_6_).

## 2. Methods

### 2.1. Participants

Taking into account the COVID-19 pandemic at the time of the study, an online survey was promoted on various gaming social media groups to recruit gamers. More specifically, a member of the research team, after obtaining the required consent, sent recruitment information about the survey to various gamer groups active on social media (primarily on Facebook). These gamer groups included players of different game genres. There was also a request in the recruitment advert to forward the link to the survey to other gamers and gamer groups. All participants were informed about the aims of the study. Additionally, all participants were informed that participation in the study was voluntary and that all data were confidential and anonymous. The final sample comprised 652 gamers (233 females; M = 28.77 years; SD = 7.18; age range: 18–48 years). The participants’ characteristics are shown in Table 1. The study was conducted in accordance with the Declaration of Helsinki, and the research team’s Ethical Committee approved the study. The dataset from the present study is available from the John Paul II Catholic University of Lublin repository database (access link: http://hdl.handle.net/20.500.12153/3433).

### 2.2. Measures

The Internet Gaming Disorder Scale-Short-Form (IGDS9-SF) [40] (Polish version: [41]) was used to assess GD criteria based on the APA framework. The scale comprises nine items (e.g., *“Do you systematically fail when trying to control or cease your gaming activity?”*) to which participants respond using a five-point scale, from 1 (*strongly disagree*) to 5 (*strongly agree*). Higher scores indicate greater risk of GD. The scale has good psychometric properties with a Cronbach’s alpha of 0.92 in the present study.

The Motives for Online Gaming Questionnaire (MOGQ) [30] (Polish version: [42]) was used to assess motives for gaming. The scale comprises 27 items (e.g., *“I play online games because gaming helps me escape reality”*), to which participants respond on a five-point scale from 1 (*almost never/never*) to 5 (*almost always/always*). Moreover, the MOGQ comprises seven subscales corresponding to seven motives for gaming: (i) escape, (ii) coping, (iii) fantasy, (iv) skill development, (v) recreation, (vi) competition, and (vi) social. A higher score on each subscale indicates greater motivation for that specific motive. The scale has good psychometric properties with following Cronbach’s alpha in the present study: 0.89 for escape; 0.81 for coping; 0.89 for fantasy; 0.90 for skill development; 0.86 for recreation; 0.89 for competition; and 0.87 for social.

The Brief Self-Control Scale (BSCS) [43] (Polish version: [44]) was used to assess self-control levels. The scale comprises 13 items (e.g., *“I am good at resisting temptation”*), to which participants respond using a five-point scale from 1 (*not at all like me*) to 4 (*very much like me*). A higher score indicates greater self-control. The scale has good psychometric properties with a Cronbach’s alpha of 0.85 in the present study.

The Patient Health Questionnaire-9 (PHQ-9) [45] (Polish version: [46]) was used to assess the risk of depressive disorder. It comprises nine items (e.g., *“Feeling tired or having little energy?”*), to which answers are given on a four-point scale from 0 (*not at all*) to 3 (*nearly every day*). A higher score indicates greater depressive symptoms. The scale has good psychometric properties with a Cronbach’s alpha of 0.88 in the present study.

The De Jong Gierveld Loneliness Scale [47] (Polish version: [48]) was used to assess loneliness. The scale comprises 11 items (e.g., *“I miss having people around me”*), to which participants respond using a five-point response scale from 1 (*definitely yes*) to 5 (*definitely no*). A higher score indicates a greater feeling of loneliness. The scale has good psychometric properties with a Cronbach’s alpha of 0.85 in the present study.

Additionally, questions were asked about demographic information (age, gender, residence, marital status), the number of hours playing videogames per week, and the gaming platform(s) used to play videogames.

### 2.3. Statistical Analysis

In order to analyze the differences between male and female gamers regarding sociodemographic and gaming platform variables, the chi-square (χ^2^) test was used. Cramér’s V [49] and φ [50] were used to assess effect size for the χ^2^ test. In order to assess the differences between male and female gamers in the number of hours playing videogames, motives for gaming, depressive symptoms, loneliness, and self-control while taking into account the non-normal distribution of some analyzed variables, the Mann–Whitney two-sample tests were used [51]. Moreover, η^2^ [50] was used to assess the effect size for the Mann–Whitney two-sample tests.

In order to explore the relationship between gaming motives, gaming disorder, depressive symptoms, loneliness, and self-control among male and female gamers, correlation analysis using rho Spearman correlation coefficient was performed. The correlation analysis was performed separately for each group and the whole sample. Then, path analysis was used to extend the analysis of relationships between the analyzed variables among male and female gamers. More specifically, path analysis with the maximum likelihood method was carried out to analyze the indirect effects between depressive symptoms, loneliness, self-control, and gaming disorder via gaming motives. 

Taking into account previous research reporting a negative relationship between disordered gaming and age [16], the relationship between age and gaming disorder was included in the model. The path model included the relationship between depressive symptoms, loneliness, self-control, and age and the relationship between gaming motives residuals. However, for clarity, these relationships are not presented in the figures see (Supplementary Material). 

Additionally, considering the violation of the multivariate normality assumption [52] (female gamers: χ^2^ [df = 24] = 422.29; *p* < 0.001; male gamers: χ^2^ [df = 24] = 454.38; *p* < 0.001), the robust standard errors and Sattora–Bentler adjustment [53] was used. The following fit indices were applied as measures of model fit in the path analysis: χ^2^, Standardized Root Means Squared Residual (SRMR), root mean square error of approximation (RMSEA), Tucker–Lewis Index (TLI), and Comparative Fit Index (CFI) [54]. The good model fits the data when RMSEA and SRMR are lower than 0.08 and CFI and TLI values are higher than 0.90 [54,55].

In order to analyze the indirect effects between depressive symptoms, loneliness, self-control, and gaming disorder via gaming motives, Zhao et al.’s [56] approach comprising the Monte Carlo method (5000 samples) to estimate standardized indirect effects with a 95% confidence interval [57] was conducted. Additionally, in order to analyze the potential differences between female (*n* = 233) and male gamers (*n* = 419) in regression weights, the Wald test [58] was used. The statistical analyses were conducted using the *IBM SPSS 27* software for descriptive statistics and correlation analysis and *Stata 14* with *medsem.ado* package [57] for path analysis see [59].

## 3. Results

There was a difference between female and male gamers in residence and marital status. However, taking into account Rea and Parker’s [60] guidelines, the effect sizes of these differences were weak. Additionally, female gamers used the following gaming platforms more often than male gamers: laptops, tablets, and smartphones. In contrast, compared to female gamers, the male gamers more often used a desktop computer, home console, and portable console for gaming see (Table 2). The effect sizes of these differences ranged from negligible to moderate (see [60]). There were also differences between male and female gamers in all motives for gaming except for escape. More specifically, male gamers showed higher levels of these motives than female gamers. However, it should be noted that the effect sizes were weak. Additionally, the female gamers had a lower level of gaming disorder and spent fewer hours playing videogames per week than male gamers. There were no gender differences in depressive symptoms, loneliness, and self-control (see Table 2).

The correlation analysis results are shown in Table 3. For female gamers, there was a positive relationship between all gaming motives, gaming hours per week, depressive symptoms, loneliness, and GD. Additionally, disordered gaming was negatively associated with self-control. Analogous results were obtained for male gamers, except for the lack of a statistically significant relationship between the recreation motive for gaming and GD. A negative correlation between age and GD was also demonstrated among male gamers. The detailed results of the correlation analysis are presented in Table 3.

The path analysis results showed that the model satisfied all fit indicators: χ^2^(df = 14) = 23.01, *p* = 0.060, SRMR = 0.023, RMSEA = 0.044, TLI = 0.973, and CFI = 0.997. For male gamers, GD was positively associated with the following gaming motives: social (β = 0.14, *p* = 0.004), fantasy (β = 0.14, *p* = 0.001), and escape (β = 0.19, *p* < 0.001). Additionally, there was a positive association between depressive symptoms (β = 0.33, *p* < 0.001), loneliness (β = 0.06, *p* = 0.036), and GD. Self-control was negatively associated with GD (β = −0.15, *p* < 0.001). Depressive symptoms were positively associated with the following gaming motives: social (β = 0.19, *p* = 0.002), competition (β = 0.13, *p* = 0.040), skill development (β = 0.17, *p* = 0.005), fantasy (β = 0.21, *p* < 0.001), escape (β = 0.32, *p* < 0.001), and coping (β = 0.28, *p* < 0.001). 

**Figure 1:**
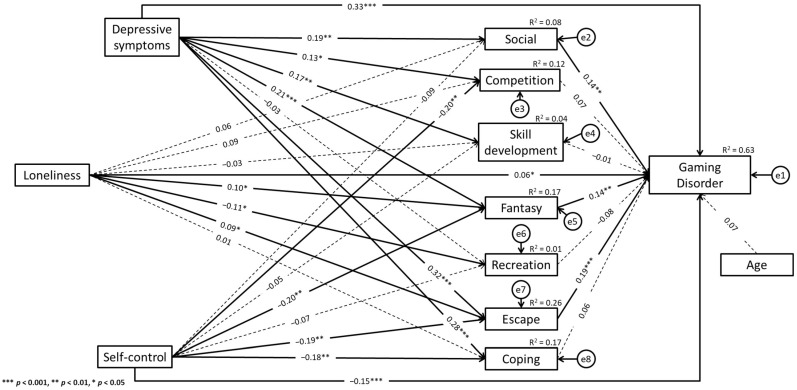
Paths model of the relationship between variables in male gamers group.

**Figure 2:**
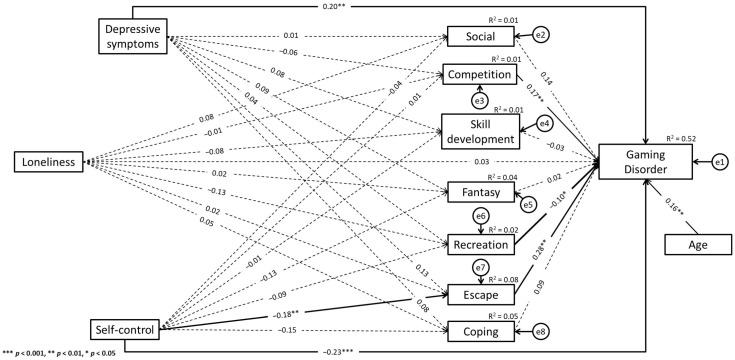
Paths model of the relationship between variables in female gamers group.

Additionally, self-control was negatively associated with the following gaming motives: competition (β = −0.20, *p* = 0.003), fantasy (β = −0.20, *p* = 0.001), escape (β = −0.19, *p* = 0.002), and coping (β = −0.18, *p* = 0.004). There was a negative association between loneliness and the recreation motive for gaming (β = −0.11, *p* = 0.028). In addition, loneliness was positively associated with fantasy (β = 0.10, *p* = 0.042) and escape (β = 0.09, *p* = 0.046). Detailed results are shown in Figure 1. For female gamers, GD was positively associated with the motives of competition (β = 0.17, *p* = 0.002) and escape (β = 0.28, *p* = 0.003). GD was positively associated with depressive symptoms (β = 0.20, *p* = 0.001) and age (β = 0.16, *p* = 0.001). Additionally, there was a negative association between recreation (β = −0.10, *p* = 0.015), self-control (β = −0.23, *p* < 0.001), and GD. Self-control was negatively associated with the escape motive for gaming (β = −0.18, *p* = 0.038). Detailed results are shown in Figure 2.

Based on the indirect effects analyses framework [56,57], there was a significant indirect effect between depressive symptoms and GD via the social motive for gaming. Additionally, depressive symptoms exerted a significant indirect effect on GD via the fantasy motive for gaming. There was also a significant indirect effect between depressive symptoms and GD via the escape motive for gaming. Considering the statistically significant direct effects between depressive symptoms and GD, these results may indicate partial mediations (see [56]). Moreover, self-control exerted a significant indirect effect on GD via the fantasy motive for gaming, and self-control also exerted a significant indirect effect on GD via the escape motive for gaming. There was a significant direct effect between self-control and GD. Consequently, these results may indicate a partial mediation (see [56]). It should be noted that the statistically significant indirect effects occurred among male gamers only (see Table 4).

The difference analysis results using the Wald test see (Table 5) showed regression weight differences between male and female gamers in the case of the relationship between GD and age. More specifically, the standardized regression weight was significant among female gamers (β = 0.16, *p* = 0.001), whereas this path was not statistically significant among male gamers (β = 0.02, *p* = 0.525). Additionally, in the case of the relationship between depressive symptoms and the escape motive for gaming and between depressive symptoms and the coping motive for gaming, a significant difference between the groups was found. More specifically, the standardized regression weights were significant among male gamers (escape motive: β = 0.32, *p* < 0.001; coping motive: β = 0.28, *p* < 0.001), whereas these paths were not statistically significant among female gamers (escape motive: β = 0.13, *p* = 0.111; coping motive: β = 0.08, *p* = 0.349). The other differences between female and male gamers in analyzed paths were not statistically significant (see Figure 1 and Figure 2).

## 4. Discussion

The present study examined the relationship between GD, motives for gaming, and psychological problems in daily life such as depressive symptoms, loneliness, and self-control deficits. More specifically, the purpose of the present study was to analyze the indirect effects between GD and psychological problems in daily life via motives for gaming among male and female Polish gamers. Additionally, the present study explored the difference between male and female gamers in motives for gaming and the relationship between GD, depressive symptoms, loneliness, self-control, and motives for gaming.

The present study showed an indirect effect between (i) depressive symptoms and GD via the escape motive for gaming and (ii) self-control and GD via the escape motive for gaming. However, these indirect effects were only found among male gamers. Consequently, H_1_ was only supported among male gamers and in the case of depressive symptoms and self-control, although the indirect effects were opposite. More specifically, there was a positive relationship between depressive symptoms and escape motive for gaming and between this motive and GD. By contrast, there was a negative relationship between self-control and escape motive for gaming and a positive relationship between this motive and GD. 

According to the compensatory internet use model [29], it is posited that gamers who are unable manage real-life situations or have difficulties with meeting their needs (e.g., need for social contact) use gaming in order to moderate negative emotions and compensate their needs through this activity. Here, depressive symptoms and self-control deficits may promote the escape motive in the development of GD. More specifically, gamers who have emotional deficits and/or have difficulties with their ability to behave in relative autonomy from external pressures, automatic cognitive functions, and/or impulses may see gaming as an escape to their real-world difficulties. However, based on the present findings, this may apply to male gamers only. Moreover, it should be noted that many previous studies examining the relationship between the escape motive and GD had highly imbalanced gender distributions with heavily male-dominated samples (see [61]). Therefore, female gamers may have a slightly different relationship pattern between psychological problems in daily life, escape motives, and GD. This is supported by the results of previous studies indicating gender differences in GD risk factors [10,24,37,38,62]. 

The present study found no indirect effects between the psychological problems and GD via motives for gaming, such as competition (H_2_), skill development (H_3_), and recreation (H_4_). One possible explanation may be related to cultural differences (in that the present study, all the gamers were Polish). More specifically, previous studies, e.g., [63,64], showed a different pattern of relationships between various predictors and problematic internet use in European countries. Consequently, various motives may be more dominant in different cultures and groups of gamers. In this context, more research is needed. However, it should be noted that among male gamers, there was an indirect effect between (i) depressive symptoms and GD via the fantasy motive for gaming and (ii) self-control and GD via the fantasy motive for gaming. According to the compensatory internet use model [29], it is posited that gamers may consider gaming as an opportunity for individuals to break away from their usual identity, try new identities in another world, and try things that cannot be done in real life (fantasy motive). According to the I-PACE model [8,9], depression and self-control deficits may contribute to GD development. Additionally, according to the present study, these factors may also be associated with the fantasy motive for gaming, which may additionally enhance the tendency toward GD.

The results of the present study showed that the depressive symptoms exerted a significant indirect effect on GD via the social motive for gaming. Given that two symptoms characterize depression—anhedonia and depressed mood [65]—it can be assumed that gamers with depressive symptoms may prefer gaming as a source of safe social contacts. Gamers with anhedonia and depressed mood may not be motivated to interact face-to-face in the real world. Consequently, they can realize their need for social contact by interacting with other gamers during the game. However, this kind of activity may deepen isolation from the real world and social contacts in the real world. Consequently, the social motive for gaming can lead to increased engagement in gaming and, in turn, to the development of GD.

H_5_ was generally supported because there were gender differences in all the analyzed gaming motives except for escape motives (see Table 2). In contrast to a previous study [31], the findings here demonstrated higher motives for gaming among male gamers than female gamers. Therefore, the present results aligned with those of Laconi et al. [34]. More specifically, as in the present study, Laconi et al. [34] reported a higher level of motives for gaming such as social, competition, skill development, fantasy, and recreation among male gamers compared to female gamers. In both the present study and that by Laconi et al. [34], there was no difference between males and females in the escape motive for gaming. The differences between these studies may be due to the sampling methods. In the study by Király et al. [31], the research recruitment was targeted online gaming websites, mostly action videogames. Taking into account the ratio of male to female gamers (approximately 9:1) and the positive relationship between action videogame use and GD among female gamers [37], the sample of female gamers in Király et al.’s [31] study may have included gamers manifesting higher scores for GD than for the population of female gamers. 

In this context, it should be noted that a meta-analysis by Su et al. [66] showed that higher levels of GD exist among males than among females. In contrast, Király et al. [31] found no differences between male and female gamers in GD, which may support the assumption of a specific sample of female gamers in the previous study [31]. The higher level of GD among female gamers (relative to the population of the female gamers), the overrepresentation of male gamers, and the positive association between motives for gaming and GD (see [32,33,34]) may have contributed to the higher levels of motives for gaming among female gamers than among the male gamers in Király et al.’s [31] study.

The present study’s findings showed that indirect effects of psychological problems in daily life such as depressive symptoms and self-control deficits via escape, fantasy, and social motives for gaming were found only among male gamers. Among female gamers, there were no analogous relationships. Additionally, there were statistically significant gender differences in the relationship between depressive symptoms and the escape motive for gaming and between depressive symptoms and the coping motive. More specifically, these relationships were statistically significant among male gamers, whereas they were statistically non-significant among female gamers. In this context, it should be noted that males often cope with depressive symptoms through maladaptive behaviors such as substance abuse, infidelity, and focusing excessively on work (see [67]). Consequently, it appears that males may treat gaming as a form of escape from problems and a means of coping with difficult situations. 

However, escaping into the virtual world does not solve the difficulties that arise in real life, which increase over time. Consequently, male gamers may spend more and more time gaming, leading to GD in some cases. Conversely, females may manifest a different form of coping with depressive symptoms than males (see [68]). Taken together, these results support the hypothesis of gender differences in the relationship between psychological problems in daily life and GD via motives for gaming (H_6_). However, further research is needed to analyze the differences between female and male gamers in these relationships more thoroughly.

It should be noted that there was weak positive relationship between loneliness and GD among male gamers only. Previous studies, e.g., [18,23,26], have shown a positive relationship between loneliness and GD. However, Cudo et al. [27] reported that the relationship between loneliness and GD was fully mediated by personal distress. Consequently, it is important to investigate to the question of whether loneliness takes an independent role in the development of GD, or whether it is dependent on other factors related to GD.

In addition, it should be noted that the present study showed gender differences in the gaming platform. More specifically, female gamers more often used laptops, tablets, and smartphones as gaming platforms compared to male gamers. In contrast, male gamers more often used desktop computers, home consoles, and portable consoles as gaming platforms compared to female gamers. Consequently, male and female gamers may manifest different gaming patterns related to the platforms used. More frequent use of mobile devices may contribute to easier videogame access than desktop devices. On the other hand, the use of desktop devices may be associated with longer gaming sessions and gaming planning than the use of mobile devices. However, future research is needed on the relevance of gaming platforms in patterns of female and male gaming.

### Limitations

The present findings should be interpreted in light of several limitations. Firstly, the study was cross-sectional and utilized self-report methods. Consequently, causal relationships cannot be determined from the data collected. Additionally, there are well-known methods biases when participants answer such questions (e.g., social desirability). Secondly, the study group was the Polish population. Consequently, it is important to be cautious when generalizing results to other countries and cultural contexts. Thirdly, considering the age range of the participants (18 to 48 years), it is important to be cautious when generalizing results to other age groups such as children and older gamers. Fourthly, participant recruitment utilized a snowballing method and by contacting gaming groups on social networks. Therefore, the participants surveyed may reflect a sample of gamers who used social media as opposed to gamers who did not have social media accounts. Finally, some demographic information about the gamers was not collected (such as their educational background and income) and whether or not they were professional gamers.

## 5. Conclusions

The present study showed there was an indirect effect between (i) depressive symptoms and GD via the escape motive for gaming and (ii) self-control and GD via the escape motive for gaming. Analogously, there was an indirect effect between (i) depressive symptoms and GD via the fantasy motive for gaming and (ii) self-control and GD via fantasy motive for gaming. Additionally, the results indicated that depressive symptoms exerted a significant indirect effect on GD via the social motive for gaming. However, it should be noted that these indirect effects were found among male gamers only. The present study also showed gender differences in all analyzed gaming motives except escape motives for gaming. More specifically, female gamers presented lower level of motives for gaming than male gamers. Consequently, the present study’s results may indicate a different pattern of relationship between GD, motives for gaming, and psychological problems in daily life such as depressive symptoms, loneliness, and self-control deficits among female gamers compared to male gamers.

## Figures and Tables

**Table 1:** Sample characteristics.

Variable	Category	Sample (*N* = 652)	
		N	Percent
Gender	Female	233	35.74
	Male	419	64.26
Residence	Small city (up to 20,000)	46	7.06
	Medium city (between 20,000 and 100,000 residents)	95	14.56
	Large city (above 100,000 residents)	402	61.66
	Village	109	16.72
Marital status	Single	227	34.82
	In a relationship	198	30.37
	Married	215	32.98
	Divorced	9	1.38
	Widowed	3	0.46
Gaming platform: Desktop computer	Yes	247	37.88
	No	405	62.12
Gaming platform: Laptop	Yes	304	46.63
	No	348	53.37
Gaming platform: Tablet	Yes	105	16.10
	No	547	83.90
Gaming platform: Home console	Yes	299	45.86
	No	353	54.14
Gaming platform: Smartphone	Yes	462	70.86
	No	190	29.14
Gaming platform: Portable console	Yes	160	24.54
	No	492	75.46

**Table 2:** Differences between female and male gamers in socio-demographic variables, gaming platform, gaming disorder, motives for gaming, depression symptoms, loneliness, and self-control.

**Variables**	**Category**	**Female** **(*N* = 233)**		**Male** **(*N* = 419)**		**χ^2^**	***p***	**Cramer’s V**
		***N***	**Percent**	***N***	**Percent**			
Residence	Small city	10	4.29	36	8.59	13.78	0.003	0.15
	Medium city	34	14.59	61	14.56			
	Large city	135	57.94	267	63.72			
	Village	54	23.18	55	13.13			
Marital status	Single	97	41.63	130	31.03	11.88	0.018	0.14
	In a relationship	71	30.47	127	30.31			
	Married	61	26.18	154	36.75			
	Divorced	4	1.72	5	1.19			
	Widowed	0	0.00	3	0.72			
**Gaming Platform**	**Category**	**Female** **(*N* = 233)**		**Male** **(*N* = 419)**		**χ^2^**	***p***	**φ**
		***N***	**Percent**	***N***	**Percent**			
Desktop computer	No	173	74.25	232	55.37	22.68	0.001	0.19
	Yes	60	25.75	187	44.63			
Laptop	No	110	47.21	238	56.80	5.34	0.019	0.09
	Yes	123	52.79	181	43.20			
Tablet	No	184	78.97	363	86.63	6.51	0.011	0.10
	Yes	49	21.03	56	13.37			
Stationary console	No	177	75.97	176	42.00	69.56	0.001	0.33
	Yes	56	24.03	243	58.00			
Smartphone	No	39	16.74	151	36.04	27.01	0.001	0.20
	Yes	194	83.26	268	63.96			
Portable console	No	203	87.12	289	68.97	26.64	0.001	0.20
	Yes	30	12.88	130	31.03			
**Variables**	**Category**	**Female** **(*N* = 233)**		**Male** **(*N* = 419)**		**z**	***p***	**η^2^**
		**M**	**SD**	**M**	**SD**			
Motives for gaming	Social	1.76	1.01	1.98	1.07	−3.48	0.001	0.02
	Escape	2.27	1.29	2.33	1.19	−1.34	0.179	0.01
	Competition	2.24	1.22	2.58	1.22	−3.72	0.001	0.02
	Coping	2.42	1.14	2.71	1.06	−3.40	0.001	0.02
	Skill development	2.58	1.25	2.83	1.23	−2.47	0.014	0.01
	Fantasy	2.14	1.26	2.37	1.24	−2.76	0.006	0.01
	Recreation	3.68	1.19	4.07	0.99	−4.01	0.001	0.02
Gaming disorder		13.79	6.72	16.47	8.00	−5.50	0.001	0.05
Gaming hours per week		7.12	9.34	12.48	11.75	−8.42	0.001	0.11
Depressive symptoms		6.57	5.90	5.94	5.38	−0.89	0.374	0.01
Loneliness		30.46	9.12	29.74	9.26	−1.22	0.224	0.01
Self-control		3.34	0.73	3.25	0.68	−1.18	0.238	0.01

Note: φ (phi) assesses the effect sizes for tests of independence in 2 × 2 contingency tables; η^2^ (eta square) assesses the effect sizes for the Mann–Whitney two-sample tests.

**Table 3:** Correlation analysis results matrix.

**Whole Sample (*N* = 652)**													
**Variables**		**(1)**	**(2)**	**(3)**	**(4)**	**(5)**	**(6)**	**(7)**	**(8)**	**(9)**	**(10)**	**(11)**	**(12)**
Motives for gaming	(1) Social												
	(2) Escape	0.46 ***											
	(3) Competition	0.53 ***	0.45 ***										
	(4) Coping	0.50 ***	0.73 ***	0.56 ***									
	(5) Skill development	0.53 ***	0.44 ***	0.58 ***	0.65 ***								
	(6) Fantasy	0.54 ***	0.72 ***	0.46 ***	0.66 ***	0.54 ***							
	(7) Recreation	0.16 ***	0.19 ***	0.29 ***	0.43 ***	0.40 ***	0.24 ***						
(8) Gaming disorder		0.42 ***	0.61 ***	0.41 ***	0.56 ***	0.34 ***	0.55 ***	0.12 **					
(9) Gaming hours per week		0.34 ***	0.26 ***	0.24 ***	0.34 ***	0.29 ***	0.33 ***	0.32 ***	0.40 ***				
(10) Depressive symptoms		0.14 ***	0.39 ***	0.11 **	0.29 ***	0.12 **	0.26 ***	−0.03	0.53 ***	0.10 **			
(11) Loneliness		0.12 **	0.20 ***	0.10 *	0.12 **	0.01	0.16 ***	−0.12 **	0.27 ***	−0.01	0.31 ***		
(12) Self-control		−0.18 ***	−0.37 ***	−0.19 ***	−0.30 ***	−0.10 *	−0.30 ***	0.01	−0.53 ***	−0.18 ***	−0.59 ***	−0.32 ***	
(13) Age		−0.08 *	−0.11 **	−0.06	−0.03	0.01	−0.08 *	−0.02	−0.04	−0.01	−0.14 ***	−0.07	0.12 **
**Female gamers (*N* = 233)**													
**Variables**		**(1)**	**(2)**	**(3)**	**(4)**	**(5)**	**(6)**	**(7)**	**(8)**	**(9)**	**(10)**	**(11)**	**(12)**
Motives for gaming	(1) Social												
	(2) Escape	0.60 ***											
	(3) Competition	0.50 ***	0.47 ***										
	(4) Coping	0.61 ***	0.81 ***	0.54 ***									
	(5) Skill development	0.49 ***	0.54 ***	0.57 ***	0.69 ***								
	(6) Fantasy	0.64 ***	0.82 ***	0.47 ***	0.77 ***	0.57 ***							
	(7) Recreation	0.14 *	0.31 ***	0.37 ***	0.44 ***	0.35 ***	0.30 ***						
(8) Gaming disorder		0.45 ***	0.59 ***	0.36 ***	0.58 ***	0.38 ***	0.53 ***	0.19 **					
(9) Gaming hours per week		0.40 ***	0.44 ***	0.32 ***	0.48 ***	0.35 ***	0.45 ***	0.46 ***	0.44 ***				
(10) Depression symptoms		0.07	0.26 ***	−0.05	0.21 **	0.07	0.18 **	0.03	0.47 ***	0.09			
(11) Loneliness		0.09	0.13 *	−0.03	0.12	−0.04	0.10	−0.10	0.21 **	0.01	0.32 ***		
(12) Self-control		−0.14 *	−0.31 ***	−0.01	−0.26 ***	−0.07	−0.23 **	−0.03	−0.53 ***	−0.17 *	−0.60 ***	−0.32 ***	
(13) Age		0.01	−0.03	0.07	0.05	0.11	0.00	−0.04	0.01	0.03	−0.11	−0.06	0.06
**Male gamers (*N* = 419)**													
**Variables**		**(1)**	**(2)**	**(3)**	**(4)**	**(5)**	**(6)**	**(7)**	**(8)**	**(9)**	**(10)**	**(11)**	**(12)**
Motives for gaming	(1) Social												
	(2) Escape	0.37 ***											
	(3) Competition	0.54 ***	0.43 ***										
	(4) Coping	0.43 ***	0.67 ***	0.55 ***									
	(5) Skill development	0.55 ***	0.38 ***	0.58 ***	0.63 ***								
	(6) Fantasy	0.48 ***	0.65 ***	0.43 ***	0.58 ***	0.50 ***							
	(7) Recreation	0.14 **	0.11 *	0.22 ***	0.42 ***	0.43 ***	0.18 ***						
(8) Gaming disorder		0.37 ***	0.62 ***	0.41 ***	0.52 ***	0.30 ***	0.54 ***	0.03					
(9) Gaming hours per week		0.27 ***	0.16 **	0.16 **	0.22 ***	0.24 ***	0.24 ***	0.19 ***	0.30 ***				
(10) Depressive symptoms		0.19 ***	0.48 ***	0.23 ***	0.36 ***	0.17 ***	0.32 ***	−0.05	0.60 ***	0.12 *			
(11) Loneliness		0.15 **	0.24 ***	0.19 ***	0.13 **	0.04	0.22 ***	−0.12 *	0.34 ***	0.01	0.31 ***		
(12) Self-control		−0.20 ***	−0.41 ***	−0.31 ***	−0.33 ***	−0.11 *	−0.34 ***	0.03	−0.55 ***	−0.16 **	−0.59 ***	−0.33 ***	
(13) Age		−0.19 ***	−0.18 ***	−0.20 ***	−0.13 **	−0.10 *	−0.16 **	−0.08	−0.18 ***	−0.16 **	−0.17 ***	−0.06	0.19 ***

Note: *** *p* < 0.001, ** *p* < 0.01, * *p* < 0.05.

**Table 4:** Standardized indirect effects with 95% confidence intervals (CIs).

Female Gamers (*N* = 233)						
Pathways	Point Estimates	Standard Error	95% CIs		z	*p*
			Lower	Upper		
Depressive symptoms → social motive → GD	0.001	0.016	−0.033	0.035	0.04	0.971
Depressive symptoms → competition motive → GD	−0.010	0.015	−0.043	0.018	−0.64	0.521
Depressive symptoms → skill development motive → GD	−0.003	0.006	−0.017	0.007	−0.46	0.646
Depressive symptoms → fantasy motive → GD	0.002	0.009	−0.016	0.022	0.19	0.849
Depressive symptoms → recreation motive → GD	−0.004	0.008	−0.022	0.013	−0.42	0.676
Depressive symptoms → escape motive → GD	0.035	0.026	−0.007	0.094	1.33	0.185
Depressive symptoms → coping motive → GD	0.006	0.012	−0.012	0.036	0.54	0.591
Self-control → social motive → GD	−0.006	0.016	−0.044	0.023	−0.40	0.689
Self-control → competition motive → GD	0.002	0.016	−0.031	0.035	0.10	0.919
Self-control → skill development motive → GD	0.001	0.005	−0.010	0.013	0.11	0.916
Self-control → fantasy motive → GD	−0.003	0.012	−0.030	0.020	−0.23	0.820
Self-control → recreation motive → GD	0.009	0.010	−0.007	0.031	0.90	0.369
Self-control → escape motive → GD	−0.050	0.030	−0.117	−0.002	−1.65	0.100
Self-control → coping motive → GD	−0.013	0.017	−0.054	0.013	−0.79	0.429
Loneliness → social motive → GD	0.011	0.012	−0.007	0.040	0.88	0.381
Loneliness → competition motive → GD	−0.001	0.014	−0.029	0.026	−0.08	0.934
Loneliness → skill development motive → GD	0.003	0.006	−0.007	0.017	0.48	0.628
Loneliness → fantasy motive → GD	0.001	0.006	−0.011	0.013	0.06	0.953
Loneliness → recreation motive → GD	0.013	0.009	−0.001	0.033	1.41	0.158
Loneliness → escape motive → GD	0.004	0.019	−0.034	0.045	0.23	0.821
Loneliness → coping motive → GD	0.004	0.009	−0.012	0.027	0.41	0.680
**Male Gamers (*N* = 419)**						
**Depressive symptoms → social motive → GD**	**0.026**	**0.013**	**0.005**	**0.055**	**2.01**	**0.044**
Depressive symptoms → competition motive → GD	0.008	0.007	−0.002	0.025	1.17	0.243
Depressive symptoms → skill development motive → GD	−0.002	0.008	−0.019	0.013	0.82	0.819
**Depressive symptoms → fantasy motive → GD**	**0.029**	**0.012**	**0.009**	**0.057**	**2.10**	**0.016**
Depressive symptoms → recreation motive → GD	0.002	0.006	−0.009	0.016	0.39	0.693
**Depressive symptoms → escape motive → GD**	**0.059**	**0.019**	**0.025**	**0.101**	**3.05**	**0.002**
Depressive symptoms → coping motive → GD	0.016	0.015	−0.012	0.046	1.05	0.295
Self-control → social motive → GD	−0.013	0.010	−0.036	0.003	−1.30	0.194
Self-control → competition motive → GD	−0.013	0.010	−0.035	0.003	−1.37	0.171
Self-control → skill development motive → GD	0.001	0.004	−0.007	0.009	0.14	0.888
**Self-control → fantasy motive → GD**	**−0.028**	**0.012**	**−0.056**	**−0.008**	**−2.26**	**0.024**
Self-control → recreation motive → GD	0.005	0.006	−0.004	0.020	0.90	0.368
**Self-control → escape motive → GD**	**−0.035**	**0.015**	**−0.069**	**−0.010**	**−2.33**	**0.020**
Self-control → coping motive → GD	−0.010	0.010	−0.033	0.008	−0.99	0.323
Loneliness → social motive → GD	0.008	0.008	−0.006	0.027	0.99	0.321
Loneliness → competition motive → GD	0.006	0.006	−0.002	0.019	1.06	0.288
Loneliness → skill development motive → GD	0.001	0.003	−0.005	0.007	0.10	0.919
Loneliness → fantasy motive → GD	0.014	0.008	0.001	0.033	1.65	0.099
Loneliness → recreation motive → GD	0.008	0.006	−0.001	0.022	1.38	0.168
Loneliness → escape motive → GD	0.017	0.010	0.001	0.039	1.68	0.093
Loneliness → coping motive → GD	0.001	0.004	−0.007	0/009	0.11	0.912

**Table 5:** Pairwise paths comparisons between female and male gamers.

Variable 1	Variable 2	Female *N* = 233		Male *N* = 419		Wald Test	*p*
		β	*p*	Β	*p*		
Gaming disorder	Age	0.16	0.001	0.02	0.525	6.18	0.013
Depressive symptoms	Escape motive	0.13	0.111	0.32	0.001	3.96	0.047
Depressive symptoms	Coping motive	0.08	0.349	0.28	0.001	3.99	0.046

## Data Availability

The dataset from the present study is available from the John Paul II Catholic University of Lublin repository database (accession link: http://hdl.handle.net/20.500.12153/3433).

## Supplementary Materials

The following supporting information can be downloaded at: https://www.mdpi.com/article/10.3390/ijerph191610438/s1, Table S1: Correlations between gaming motive residuals, and between self-control, loneliness, depressive symptoms and age.

## References

[1] Bediou B., Adams D.M., Mayer R.E., Tipton E., Green C.S., Bavelier D. (2018). Meta-analysis of action video game impact on perceptual, attentional, and cognitive skills. Psychol. Bull..

[2] Bergonse R. (2017). Fifty years on, what exactly is a videogame? An essentialistic definitional approach. Comput. Games J..

[3] Statista Forecast of Video Games Users by Segment in the World from 2017 to 2025. https://www.statista.com/forecasts/456610/video-games-users-in-the-world-forecast.

[4] Statista Number of Video Game Users in Poland from 2017 to 2025, by Segment. https://www.statista.com/forecasts/1221164/poland-users-video-game-market-by-segment.

[5] Cudo A., Kopiś N., Stróżak P., Zapała D. (2018). Problematic video gaming and problematic internet use among Polish young adults. Cyberpsychol. Behav. Soc. Netw..

[6] Griffiths M.D., Attrill-Smith A., Fullwood C., Keep M., Kuss D.J. (2019). The therapeutic and health benefits of playing video games. The Oxford Handbook of Cyberpsychology.

[7] Kort-Butler L.A. (2021). The well-being of gamers, video game players, and non-players. Soc. Sci. J..

[8] Brand M., Young K.S., Laier C., Wölfling K., Potenza M.N. (2016). Integrating psychological and neurobiological considerations regarding the development and maintenance of specific Internet-use disorders: An Interaction of Person-Affect-Cognition-Execution (I-PACE) model. Neurosci. Biobehav. Rev..

[9] Brand M., Wegmann E., Stark R., Müller A., Wölfling K., Robbins T.W., Potenza M.N. (2019). The Interaction of Person-Affect-Cognition-Execution (I-PACE) model for addictive behaviors: Update, generalisation to addictive behaviors beyond internet-use disorders, and specification of the process character of addictive behaviors. Neurosci. Biobehav. Rev..

[10] Kuss D.J., Kristensen A.M., Williams A.J., Lopez-Fernandez O. (2022). To be or not to be a female gamer: A qualitative exploration of female gamer identity. Int. J. Environ. Res. Public Health.

[11] Lopez-Fernandez O., Williams A.J., Griffiths M.D., Kuss D.J. (2019). Female gaming, gaming addiction, and the role of women within gaming culture: A narrative literature review. Front. Psychiatry.

[12] Stevens M.W., Dorstyn D., Delfabbro P.H., King D.L. (2021). Global prevalence of gaming disorder: A systematic review and meta-analysis. Aust. N. Z. J. Psychiatry..

[13] American Psychiatric Association (2013). Diagnostic and Statistical Manual of Mental Disorders.

[14] World Health Organization International Classification of Diseases for Mortality and Morbidity Statistics (11th Revision). https://icd.who.int/browse11/l-m/en.

[15] Cudo A., Szewczyk M., Błachnio A., Przepiórka A., Jarząbek-Cudo A. (2020). The role of depression and self-esteem in Facebook intrusion and gaming disorder among young adult gamers. Psychiatr. Q..

[16] Montag C., Schivinski B., Sariyska R., Kannen C., Demetrovics Z., Pontes H.M. (2019). Psychopathological symptoms and gaming motives in disordered gaming—A psychometric comparison between the WHO and APA diagnostic frameworks. J. Clin. Med..

[17] Ostinelli E.G., Zangani C., Giordano B., Maestri D., Gambini O., D’Agostino A., Furukawa T.A., Purgato M. (2021). Depressive symptoms and depression in individuals with internet gaming disorder: A systematic review and meta-analysis. J. Affect. Disord..

[18] Pontes H.M., Schivinski B., Sindermann C., Li M., Becker B., Zhou M., Montag C. (2021). Measurement and conceptualisation of gaming disorder according to the World Health Organization framework: The development of the Gaming Disorder Test. Int. J. Ment. Health Addict..

[19] LaRose R., Lin C.A., Eastin M.S. (2003). Unregulated internet usage: Addiction, habit, or deficient self-regulation?. Media Psychol..

[20] Teng Z., Pontes H.M., Nie Q., Griffiths M.D., Guo C. (2021). Depression and anxiety symptoms associated with internet gaming disorder before and during the COVID-19 pandemic: A longitudinal study. J. Behav. Addict..

[21] Liu Y., Gong R., Yu Y., Xu C., Yu X., Chang R., Wang H., Wang S., Wang Q., Cai Y. (2021). Longitudinal predictors for incidence of internet gaming disorder among adolescents: The roles of time spent on gaming and depressive symptoms. J. Adolesc..

[22] Jeong H., Yim H.W., Lee S.Y., Lee H.K., Potenza M.N., Jo S.J., Son H.J. (2019). Reciprocal relationship between depression and Internet gaming disorder in children: A 12-month follow-up of the iCURE study using cross-lagged path analysis. J. Behav. Addict..

[23] Krossbakken E., Pallesen S., Mentzoni R.A., King D.L., Molde H., Finserås T.R., Torsheim T. (2018). A cross-lagged study of developmental trajectories of video game engagement, addiction, and mental health. Front. Psychol..

[24] Cudo A., Misiuro T., Griffiths M.D., Torój M. (2020). The relationship between problematic video gaming, problematic Facebook use, and self-control dimensions among female and male Gamers. Adv. Cogn. Psychol..

[25] Mills D.J., Allen J.J. (2020). Self-determination theory, internet gaming disorder, and the mediating role of self-control. Comput. Hum. Behav..

[26] Lemmens J.S., Valkenburg P.M., Peter J. (2011). Psychosocial causes and consequences of pathological gaming. Comput. Hum. Behav..

[27] Cudo A., Kopiś N., Zabielska-Mendyk E. (2019). Personal distress as a mediator between self-esteem, self-efficacy, loneliness and problematic video gaming in female and male emerging adult gamers. PLoS ONE.

[28] Davis M.H. (1983). Measuring individual differences in empathy: Evidence for a multidimensional approach. J. Pers. Soc. Psychol..

[29] Kardefelt-Winther D. (2014). A conceptual and methodological critique of internet addiction research: Towards a model of compensatory internet use. Comput. Hum. Behav..

[30] Demetrovics Z., Urbán R., Nagygyörgy K., Farkas J., Zilahy D., Mervó B., Reindl A., Ágoston C., Kertész A., Harmath E. (2011). Why do you play? The development of the Motives for Online Gaming Questionnaire (MOGQ). Behav. Res. Methods.

[31] Király O., Urbán R., Griffiths M.D., Ágoston C., Nagygyörgy K., Kökönyei G., Demetrovics Z. (2015). The mediating effect of gaming motivation between psychiatric symptoms and problematic online gaming: An online survey. J. Med. Internet Res..

[32] Kircaburun K., Demetrovics Z., Griffiths M.D., Király O., Kun B., Tosuntaş Ş.B. (2020). Trait emotional intelligence and internet gaming disorder among gamers: The mediating role of online gaming motives and moderating role of age groups. Int. J. Ment. Health Addict..

[33] Kuss D.J., Louws J., Wiers R.W. (2012). Online gaming addiction? Motives predict addictive play behavior in massively multiplayer online role-playing games. Cyberpsychol. Behav. Soc. Netw..

[34] Laconi S., Pirès S., Chabrol H. (2017). Internet gaming disorder, motives, game genres and psychopathology. Comput. Hum. Behav..

[35] Kircaburun K., Jonason P.K., Griffiths M.D. (2018). The Dark Tetrad traits and problematic online gaming: The mediating role of online gaming motives and moderating role of game types. Pers. Individ. Differ..

[36] Maroney N., Williams B.J., Thomas A., Skues J., Moulding R. (2019). A stress-coping model of problem online video game use. Int. J. Ment. Health Addict..

[37] Cudo A., Torój M., Misiuro T., Griffiths M.D. (2020). Problematic Facebook use and problematic video gaming among female and male gamers. Cyberpsychol. Behav. Soc. Netw..

[38] Cudo A., Wojtasiński M., Tużnik P., Griffiths M.D., Zabielska-Mendyk E. (2020). Problematic Facebook use and problematic video gaming as mediators of relationship between impulsivity and life satisfaction among female and male gamers. PLoS ONE.

[39] McLean L., Griffiths M.D. (2019). Female gamers’ experience of online harassment and social support in online gaming: A qualitative study. Int. J. Ment. Health Addict..

[40] Pontes H.M., Griffiths M.D. (2015). Measuring DSM-5 internet gaming disorder: Development and validation of a short psychometric scale. Comput. Hum. Behav..

[41] Schivinski B., Brzozowska-Woś M., Buchanan E.M., Griffiths M.D., Pontes H.M. (2018). Psychometric assessment of the internet gaming disorder diagnostic criteria: An item response theory study. Addict. Behav. Rep..

[42] Grzegorzewska I., Cierpiałkowska L. (2018). Uzależnienia Behawioralne.

[43] Tangney J.P., Baumeister R.F., Boone A.L. (2004). High self-control predicts good adjustment, less pathology, better grades, and interpersonal success. J. Pers..

[44] Pilarska A., Baumeister R.F. (2018). Psychometric properties and correlates of the Polish version of the Self-Control Scale (SCS). Pol. Psychol. Bull..

[45] Kroenke K., Spitzer R.L., Williams J.B. (2001). The PHQ-9: Validity of a brief depression severity measure. J. Gen. Intern. Med..

[46] Kokoszka A., Jastrzębski A., Obrębski M. (2016). Psychometric properties of the Polish version of Patient Health Questionnaire-9. Psychiatria.

[47] De Jong Gierveld J., Van Tilburg T. (1999). Living arrangements of older adults in the Netherlands and Italy: Coresidence values and behaviour and their consequences for loneliness. J. Cross-Cult. Gerontol..

[48] Grygiel P., Humenny G., Rebisz S., Świtaj P., Sikorska J. (2013). Validating the Polish adaptation of the 11-item De Jong Gierveld Loneliness Scale. Eur. J. Psychol. Assess..

[49] Cramér H. (1961). Mathematical Methods of Statistics.

[50] Fritz C.O., Morris P.E., Richler J.J. (2012). Effect size estimates: Current use, calculations, and interpretation. J. Exp. Psychol. Gen..

[51] Mann H.B., Whitney D.R. (1947). On a test of whether one of two random variables is stochastically larger than the other. Ann. Math. Stat..

[52] Doornik J.A., Hansen H. (2008). An omnibus test for univariate and multivariate normality. Oxf. Bull. Econ. Stat..

[53] Satorra A., Bentler P.M., von Eye A., Clogg C.C. (1994). Corrections to test statistics and standard errors in covariance structure analysis. Latent Variables Analysis: Applications for Developmental Research.

[54] Kline R.B. (2011). Principles and Practice of Structural Equation Modelling.

[55] Hu L.T., Bentler P.M. (1999). Cutoff criteria for fit indexes in covariance structure analysis: Conventional criteria versus new alternatives. Struct. Equ. Model..

[56] Zhao X., Lynch J.G., Chen Q. (2010). Reconsidering Baron and Kenny: Myths and truths about mediation analysis. J. Consum. Res..

[57] Mehmetoglu M. (2018). Medsem: A Stata package for statistical mediation analysis. Int. J. Comput. Econ. Econom..

[58] Wald A. (1943). Tests of statistical hypotheses concerning several parameters when the number of observations is large. Trans. Am. Math. Soc..

[59] Acock A.C. (2013). Discovering Structural Equation Modeling using Stata.

[60] Rea L.M., Parker R.A. (1992). Designing and Conducting Survey Research.

[61] Melodia F., Canale N., Griffiths M.D. (2022). The role of avoidance coping and escape motives in problematic online gaming: A systematic literature review. Int. J. Ment. Health Addict..

[62] Cudo A., Dobosz M., Griffiths M.D., Kuss D.J. (2022). The relationship between early maladaptive schemas, depression, anxiety and problematic video gaming among female and male gamers. Int. J. Ment. Health Addict..

[63] Laconi S., Kaliszewska-Czeremska K., Gnisci A., Sergi I., Barke A., Jeromin F., Groth J., Gamez-Guadix M., Ozcan N.K., Demetrovics Z. (2018). Cross-cultural study of problematic internet use in nine European countries. Comput. Hum. Behav..

[64] Laconi S., Urbán R., Kaliszewska-Czeremska K., Kuss D.J., Gnisci A., Sergi I., Barke A., Jeromin F., Groth J., Gamez-Guadix M. (2019). Psychometric evaluation of the nine-item Problematic Internet Use Questionnaire (PIUQ-9) in nine European samples of internet users. Front. Psychiatry.

[65] Łojko D., Suwalska A., Rybakowski J. (2014). Bipolar and related disorders and depressive disorders in DSM-5. Psychiatr. Pol..

[66] Su W., Han X., Yu H., Wu Y., Potenza M.N. (2020). Do men become addicted to internet gaming and women to social media? A meta-analysis examining gender-related differences in specific internet addiction. Comput. Hum. Behav..

[67] Whittle E.L., Fogarty A.S., Tugendrajch S., Player M.J., Christensen H., Wilhelm K., Hadzi-Pavlovic D., Proudfoot J. (2015). Men, depression, and coping: Are we on the right path?. Psychol. Men Masc..

[68] Kleinke C.L., Staneski R.A., Mason J.K. (1982). Sex differences in coping with depression. Sex Roles.

