# Supplementary material for: The Relationship between Depressive Symptoms, Loneliness, Self-Control, and Gaming Disorder among Polish Male and Female Gamers: The Indirect Effects of Gaming Motives
Source: Int J Environ Res Public Health. 2022 Aug 22;19(16):10438. doi: 10.3390/ijerph191610438 (PMC9408588; doi:10.3390/ijerph191610438)
Supplement: Supplementary file 1 [file ijerph-19-10438-s001.zip › ijerph-1877690-supplementary.pdf]

Table S1. Correlations between gaming motive residuals, and between self-control, loneliness, depressive symptoms and age

Groups	*r*	SE	*p*≤
Social <--> Competition			
Male	0.49	0.04	0.001
Female	0.51	0.06	0.001
Social <--> Skill development			
Male	0.52	0.04	0.001
Female	0.47	0.06	0.001
Social <--> Fantasy			
Male	0.41	0.04	0.001
Female	0.60	0.06	0.001
Social <--> Recreation			
Male	0.20	0.04	0.001
Female	0.19	0.05	0.001
Social <--> Escape			
Male	0.34	0.05	0.001
Female	0.56	0.06	0.001
Social <--> Coping			
Male	0.40	0.04	0.001
Female	0.59	0.05	0.001
Competition <--> Skill development			
Male	0.56	0.04	0.001
Female	0.58	0.05	0.001
Competition <--> Fantasy			
Male	0.33	0.05	0.001
Female	0.51	0.06	0.001
Competition <--> Recreation			
Male	0.31	0.04	0.001
Female	0.39	0.05	0.001
Competition <--> Escape			
Male	0.35	0.05	0.001
Female	0.51	0.06	0.001
Competition <--> Coping			
Male	0.50	0.04	0.001
Female	0.59	0.05	0.001
Skill development <--> Fantasy			
Male	0.47	0.04	0.001
Female	0.56	0.05	0.001
Skill development <--> Recreation			
Male	0.46	0.04	0.001
Female	0.39	0.06	0.001
Skill development <--> Escape			
Male	0.36	0.05	0.001
Female	0.53	0.05	0.001
Skill development <--> Coping			
Male	0.63	0.03	0.001

Female	0.70	0.04	0.001
Fantasy <--> Recreation			
Male	0.28	0.04	0.001
Female	0.36	0.05	0.001
Fantasy <--> Escape			
Male	0.57	0.04	0.001
Female	0.79	0.03	0.001
Fantasy <--> Coping			
Male	0.53	0.04	0.001
Female	0.78	0.03	0.001
Recreation <--> Escape			
Male	0.22	0.05	0.001
Female	0.37	0.05	0.001
Recreation <--> Coping			
Male	0.48	0.03	0.001
Female	0.48	0.05	0.001
Escape <--> Coping			
Male	0.61	0.03	0.001
Female	0.80	0.03	0.001
Depressive symptoms <--> Self-control			
Male	-0.60	0.04	0.001
Female	-0.58	0.04	0.001
Depressive symptoms <--> Loneliness			
Male	0.35	0.04	0.001
Female	0.32	0.06	0.001
Depressive symptoms <--> Age			
Male	-0.19	0.04	0.001
Female	-0.07	0.06	0.276
Self-control <--> Loneliness			
Male	-0.38	0.05	0.001
Female	-0.37	0.07	0.001
Self-control <--> Age			
Male	0.19	0.04	0.001
Female	0.05	0.06	0.350
Loneliness <--> Age			
Male	-0.05	0.05	0.325
Female	-0.05	0.05	0.377
